# Proteomic profile of the dentine pellicle modified with plant polyphenols and fluoride

**DOI:** 10.1007/s00784-026-07097-y

**Published:** 2026-09-14

**Authors:** Thiago Saads Carvalho, Tommy Baumann, Marilia Afonso Rabelo Buzalaf, Daniela Rios, Matthias Hannig, Samira Helena Niemeyer

**Affiliations:** 1 Department of Restorative, Preventive and Pediatric Dentistry, School of Dental Medicine, University of Bern, Freiburgstrasse 7, Bern, CH 3010 Switzerland; 2 Department of Biological Sciences, Bauru School of Dentistry, University of São Paulo, Bauru, Brazil; 3 Department of Pediatric Dentistry, Orthodontics and Public Health, Bauru School of Dentistry, University of São Paulo, Bauru, Brazil; 4 Clinic of Operative Dentistry, Periodontology and Preventive Dentistry, Saarland University, Homburg, Germany

**Keywords:** Dentine pellicle, Saliva, Polyphenols, Plant extracts, Fluoride, Pellicle modification.

## Abstract

**Objectives:**

Despite its protective role, the dentine pellicle has rarely been studied, therefore we aimed to map out the proteomic profile of in vitro dentine pellicles before and after modification.

**Materials and methods:**

A total of 135 human dentine specimens were prepared. After initial pellicle formation with 150 µl pooled human saliva (37 °C, 30 min), the dentine specimens were immersed in one of 9 pellicle modification solutions (2 ml/specimen): deionized water (non-modified pellicle), SnCl_2_/NaF/AmF (commercial solution containing 800 ppm Sn^2+^ and 500 ppm F^−^), NaF solution (500 ppm F^−^ ), and six polyphenol solutions (2 mg / ml) with or without 500 ppm F^−^: blueberry extract (BBE and BBE + F^−^), green tea extract (GTE and GTE + F^−^) and grape seed extract (GSE and GSE + F^−^). After another aliquot of saliva (150 µl, 37 °C, 60 min), the pellicles were harvested with sodium dodecyl sulphate by rubbing with cotton balls, and taken to proteomic analyses by Liquid Chromatography-Tandem Mass Spectrometry after tryptic digestion.

**Results:**

A total of 382 proteins were identified in all the proteomic analyses for all groups. Pellicle modification with fluoride, either NaF or SnCl_2_/NaF/AmF, led to the presence of 12 or 14 exclusive proteins, respectively, whereas modification with the solutions containing polyphenols presented less exclusive proteins (4–6 proteins). The number of exclusive proteins was even lower for when polyphenols and fluoride (GTE + F^−^ and GSE + F^−^) were used, with lower abundance of proteases.

**Conclusions:**

We conclude that NaF and SnCl_2_/NaF/AmF significantly modify the proteome of the dentine pellicle. The combination of fluoride with polyphenols further lowers the abundance of proteins and proteases, which explains the positive effect of these solutions on the dentine pellicles.

**Clinical relevance:**

Plant extract solutions with fluoride can significantly modify the proteomic structure of the dentine pellicle, which clarifies the mechanism of action of polyphenols on the protection of dentine demineralization.

## Introduction

 Saliva serves as a natural protection for teeth, creating a semi-permeable acquired pellicle over the dental surfaces. This pellicle consists of proteins, glycoproteins, peptides, lipids, and various bio-macromolecules. This pellicle is of great importance in the protection of the dental surfaces. However, the majority of research has focused on the pellicle formed on enamel [1–6], while the pellicle formed on dentine [7–9] still remains a largely neglected topic [10]. This dentine pellicle is quite different from that of enamel. Because if the special histology of dentine, with its relatively larger water content and organic collagenic fibrils network, can greatly influence the dentine acquired pellicle [11].

In the last few years, pellicle studies have concentrated on its protective effect or improving this effect by modifying the proteomic content. This can be done, for example, through natural compounds and plant polyphenols [4, 12–14]. Some polyphenols have a strong affinity to specific salivary proteins [15], triggering the aggregation of these proteins onto and within the acquired pellicle. This process contributes to an increase in the thickness of the pellicle [16, 17] and, consequently, a reduction in the pellicle’s permeability to acids, leading to better protection of the underlying tooth structure against demineralization [11, 18].

Green tea and Black tea have positive effects on the enamel salivary pellicle [19]. Both teas are made from the same plant leaves (Camellia sinensis), but they have different oxidation levels, leading to considerable chemical differences between them, where green tea is rich in catechins, while black tea is rich in theaflavins [20–22]. Modification of the acquired pellicle with green tea causes thicker and more electron-dense pellicles [23, 24], with considerable modifications to its proteomic profile, containing more acid-resistant proteins like statherin [25]. In turn, this modified pellicle is significantly more protective against demineralization [26, 27].

Recently, Niemeyer et al. [28] showed that polyphenols modifying the dentine acquired pellicle will have a three-fold mode of action against demineralization. Firstly, they will interact with the collagen (organic) layer of the dentine itself and crosslink the collagen matrix. Furthermore, they secondly will bind to the matrix metalloproteinases (MMPs) present in the dentine and saliva, thus inactivating these proteins and inhibiting their breakage of the collagen layer. Finally, they thirdly will also crosslink the proteins of the acquired pellicle, thereby, increasing the protein-protein interaction and the thickness of the pellicle. If fluoride is additionally added to the polyphenols, the protective effects of the modified pellicle will be enhanced even further [28, 29]. This is because fluoride will have yet additional modes of action, as it can inhibit MMP-2 and MMP-9 [30], and likely have an effect on the composition of the pellicle. A further mode of action of fluoride is its reaction with the dentine mineral to form CaF_2_-like particles on the dentine surface, but this reaction is dependent on low pH and high fluoride concentrations [31], and therefore rather unlikely at the usual composition of rinsing solutions.

Despite all these advantages, the proteomic changes to the dentine pellicle after using solutions containing polyphenols and fluoride have not yet been fully described. The present study, therefore, presents the proteomic profile of dentine pellicles modified with fluoride solutions, solutions containing polyphenol-rich plant extracts, as well as solutions containing both polyphenols and fluoride. The null-hypothesis was that the treatments do not change the composition of the dentine pellicles.

## Materials and methods

### Specimen preparation

Human permanent molars were selected from a biobank, where the teeth had been extracted by dental practitioners in Switzerland. Before the extraction, the patients were informed about the use of their teeth for research purposes and their consent was given. Because we are using pooled teeth, the local ethics committee categorized the samples as “irreversibly anonymised”, and no previous ethical approval was necessary. The present experiment was carried out in conformity with the approved guidelines and regulations of the local ethical committee (Kantonale Ethikkommission: KEK).

A total of 135 dentine squares (4 × 4 mm) were obtained from the roots of 135 human molars that had been visually inspected to exclude superficial defect or cavitation (IsoMet Low Speed Saw, Buehler). They were ground flat with abrasive silicon carbide paper discs of decreasing grain size (18.3 μm to 5 μm), and later polished with 3 μm grain size diamond paste (DPStick P, Struers, for 60 s) under constant tap water cooling (TegraPol-15, Struers). The specimens were rinsed with deionized water in an ultrasound bath (60 s) between each step. They were kept in tap water (non-fluoridated) at 4 °C, and right before the start of the experiment, a final polishing was performed with 1 μm grain size diamond pastes (DP-Stick P, Struers, for 60 s) under constant cooling (TegraPol-15, Struers) and rinsed with deionized water in an ultrasound bath for 60 s.

### Stimulated human saliva collection

Clarified stimulated human saliva was used in the present study. The saliva was collected from adult healthy donors (non-smokers, no known medical conditions/drug use, no fixed orthodontic devices). Similarly, to teeth, the local ethical committee (KEK) considers pooled samples of saliva as irreversibly anonymized, therefore prior ethical approval was not required.

Stimulated saliva was collected in the morning (11 males and 8 females; age range 23–45; mean 26.7 years). Donors chewed on paraffin (Paraffin pellets, Ivoclar Vivadent) for 10 min and stimulated saliva (average 9.8 ml/donor) was collected into ice-chilled vials. The stimulated saliva was immediately pooled, clarification was perfomed by centrifugation (3’000 g, 15 min, 4 °C), and the clarified supernatant fraction was collected.

To avoid degradation of the salivary proteins, protease inhibitors (phenylmethanesulfonyl fluoride; n-ethylmaleimide; 1,10-phenanthroline monohydrate) were added to the saliva. These inhibitors were initially dissolved in methanol, creating a solution with a concentration of 100 mM per inhibitor. This solution was added to the clarified human saliva (1:100), resulting in saliva containing 1 mM of each of the inhibitors [32]. Then, aliquots were prepared and stored at −80 °C until the beginning of the experiment.

### Experimental procedure

The dentine specimens were divided into 9 experimental groups (*n* = 15/group):

- Deionized water (negative control, native pH, non-modified pellicle)
- SnCl_2_/NaF/AmF (positive control, pellicle modified with a commercial mouthrinse, elmex^®^ Erosion Protection containing 800 ppm Sn^2+^, as SnCl_2_, and 500 ppm F^-^, as NaF and AmF), pH 4.5
- NaF (pellicle modified with a NaF solution, 500 ppm F^-^), pH 5.8
- Polyphenol solutions (2 g polyphenols/L) either with or without 500 ppm F^-^ (as NaF), pH 5.8:
  - ○ Blueberry extract (BBE) and BBE + F^-^
  - ○ Green tea extract (GTE) and GTE + F^-^
  - ○ Grape seed extract (GSE) and GSE + F^-^.

For the experiment, dentine specimens received an aliquot (150 µl) of freshly thawed human saliva and were incubated at 37 °C for 30 min, thus allowing for initial protein adherence and basal pellicle formation. The specimens then were immersed in 2 ml of one of the experimental solutions for 2 min at 25 °C. Afterwards a fresh aliquot (150 µl) of saliva was placed on the specimens and they were incubated at 37 °C for a further 60 min.

### Pellicle harvesting and proteomic analyses

The dentine specimens containing the pellicles were washed with deionized water for 5 s and pellicles were harvested. For that, 5 µl of SDS (Sodium dodecyl-sulfate) was applied to the surface of each specimen, then the surfaces were rubbed with cotton pellets. The cotton pellets had previously been boiled in SDS for 5 min to remove possible contaminations. This was repeated 3 times, to get three biological replicates. The pellicles were then eluted from the cotton pellets by centrifugation and loaded on an acrylamide gel (20%), which was run at 120 V for 15 min. The gels were fixed. And the lanes containing the proteins were cut out and submitted to tryptic digestion and proteomic analyses with Liquid Chromatography with tandem Mass Spectrometry (LC-MS/MS). The proteomic specimens ran through the mass spectrometer in three repetitions, according to the tree biological replicates, and the identified proteins were recorded onto an excel sheet. Protein abundance was quantified by label-free quantification (LFQ) in MaxQuant. LFQ values represent normalized arbitrary intensity units, which were subsequently used to calculate fold changes (FC) between the experimental groups.

### Statistical analysis

From all identified proteins in all replicates, only the ones that were present in at least two repetitions were considered for the analyzes. Firstly, the total number of proteins identified in each of the experimental groups was considered. Then qualitative analyses were performed, where each pair of groups was compared in order to identify proteins in common in both groups. Then all groups were compared to identify the common proteins present in all three groups. The proteins identified exclusively in each experimental group were also verified. Differential of abundance was performed on label-free quantification data using the Empirical Bayes test. Significance was calculated based on a minimal log2 fold change of 1 and the p-values were adjusted with the false discovery rate-controlled Benjamini and Hochberg multiple test correction. Significantly more abundant proteins are presented with an up arrow (↑) and significantly less abundant proteins are presented with a down arrow (↓).

## Results

**Fig. 1:**
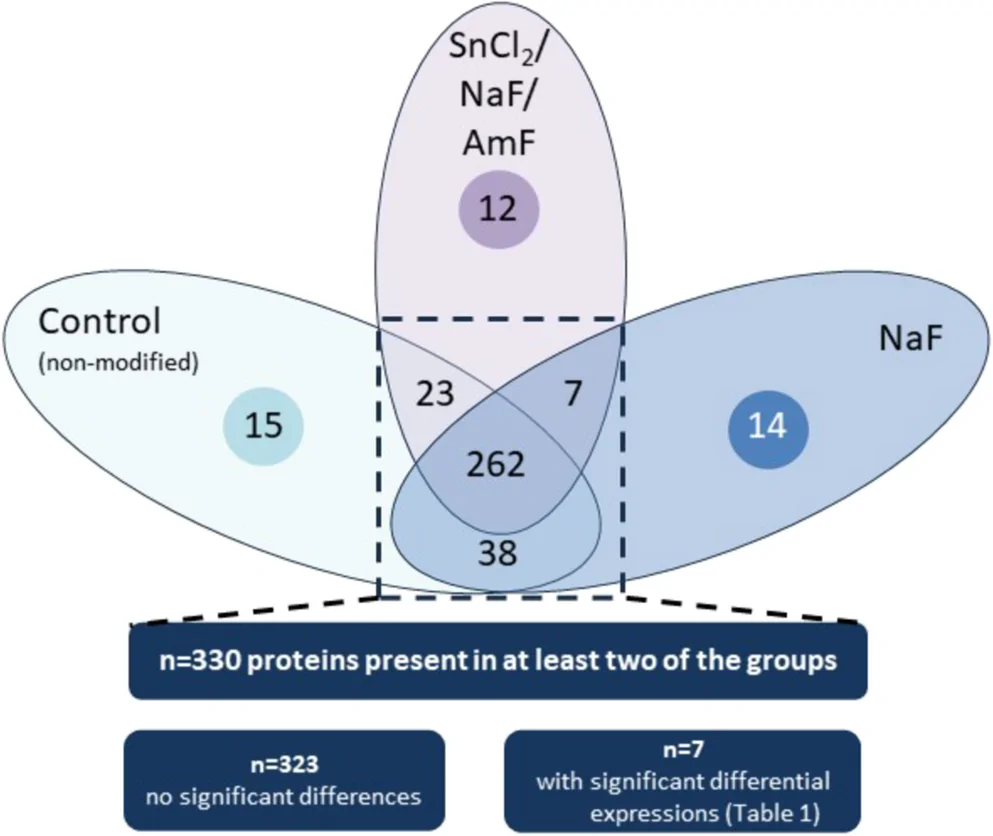
Number of exclusive proteins, as well as non-exclusive proteins with those with significantly different abundance, present in the respective groups: negative control, NaF and SnCl_2_/NaF/AmF

**Fig. 2:**
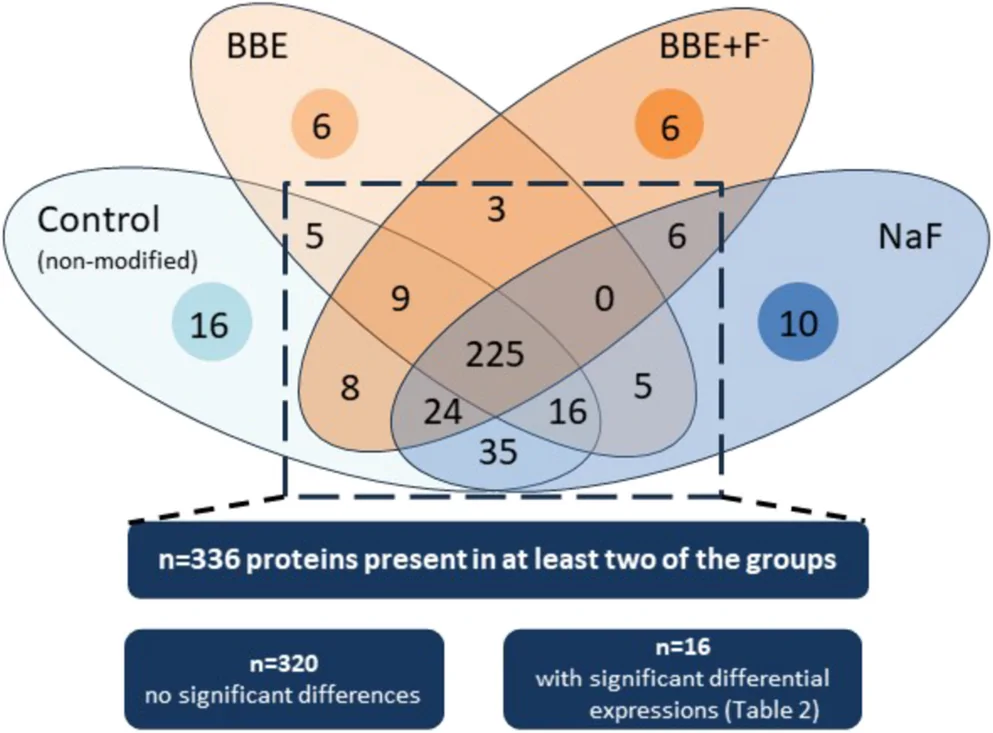
Number of exclusive proteins, as well as non-exclusive proteins with those with significantly different abundance, present in the respective groups: negative control, BBE, BBE + F^−^ and NaF

**Fig. 3:**
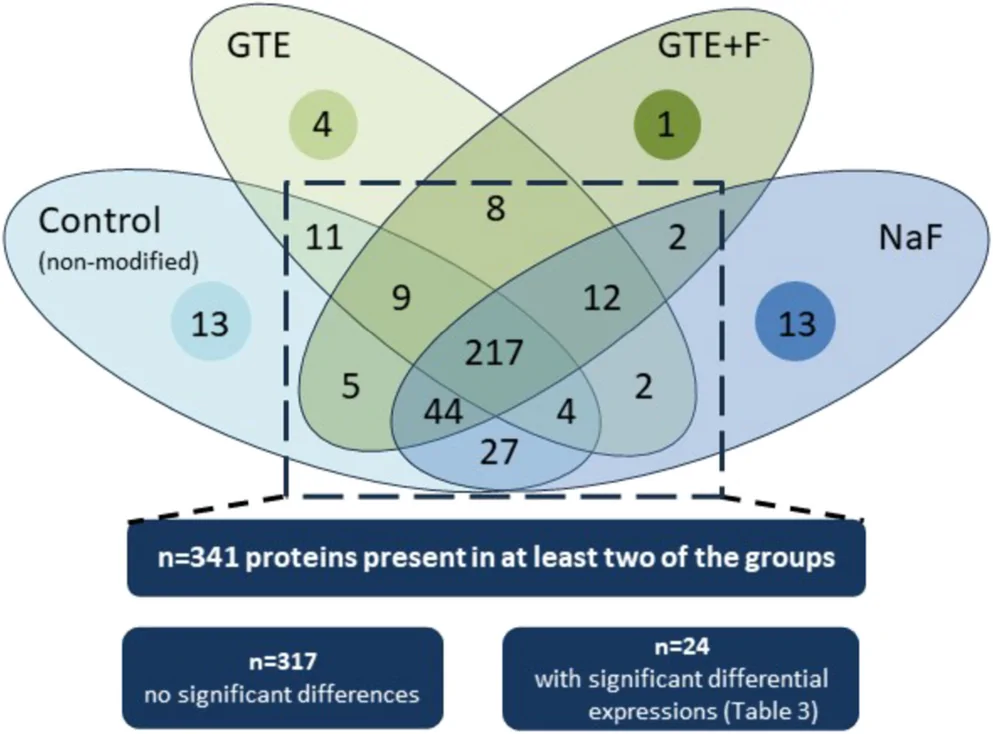
Number of exclusive proteins, as well as non-exclusive proteins with those with significantly different abundance, present in the respective groups: negative control, GTE, GTE + F^−^ and NaF

**Fig. 4:**
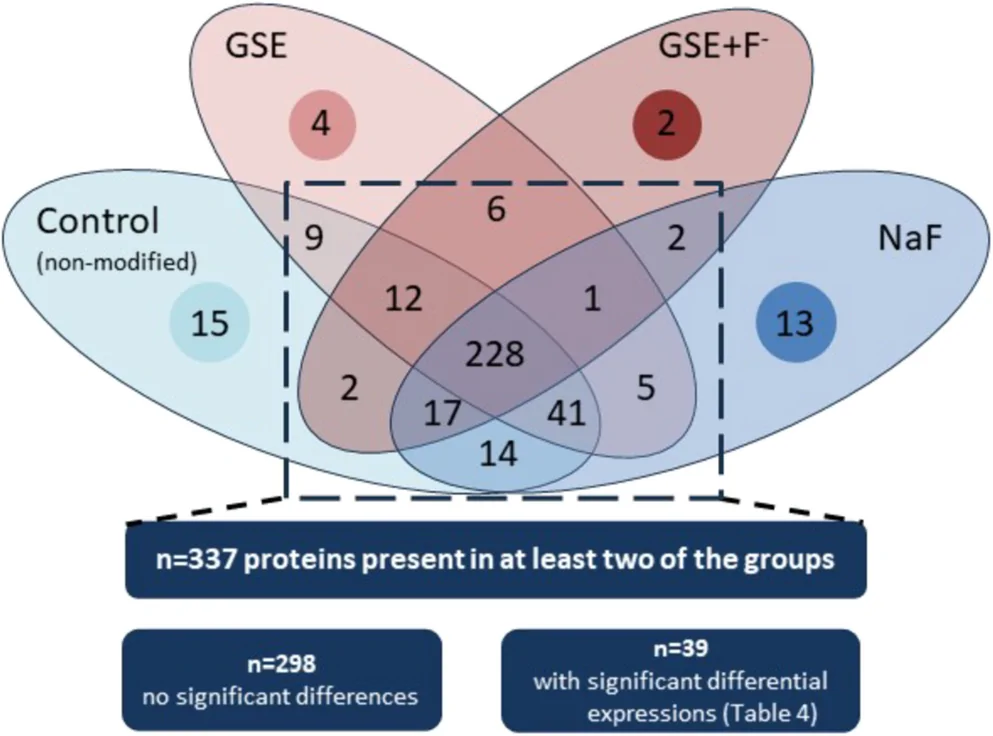
Number of exclusive proteins, as well as non-exclusive proteins with those with significantly different abundance, present in the respective groups: negative control, GSE, GSE + F^−^ and NaF

A total of 382 proteins were identified in all the proteomic analyses for all groups. The specific number of proteins in the different groups are shown in Figs. 1, 2, 3 and 4.

The non-modified pellicle contained around 13 to 16 exclusive proteins when comparing to the other modified pellicles (Figs. 1, 2, 3 and 4). Pellicle modification with fluoride, either with NaF or SnCl_2_/NaF/AmF, led to the presence of 12 and 14 exclusive proteins, respectively (Fig. 1). Pellicle modification with SnCl_2_/NaF/AmF led to lower abundance of 5 proteins (Fig. 1; Table 1).

**Table 1:** Proteins with different abundances and their biological functions in the comparison of groups: negative control, NaF and SnCl_2_/NaF/AmF (from Fig. 1)

Protein	Difference in abundance (log_2_ fold change)	Biological Function
Hemopexin	↓ in NaF compared to Control (−4.74) and SnCl_2_/NaF/AmF (−5.43)	Transport heme
Transgelin-2	↓ in NaF compared to SnCl_2_/NaF/AmF (−5.51)	Cytoskeleton organization
Protein S100-A6	↓ in SnCl_2_/NaF/AmF compared to Control (−8.39) and NaF (−7.95)	Calcium ion binding
Protein S100-A14	↓ in SnCl_2_/NaF/AmF compared to NaF (−6.91)	Cell survival and apoptosis
Immunoglobulin heavy variable 3–15	↓ in SnCl_2_/NaF/AmF compared to Control (−6.68)	Immunity
Glucosidase 2 subunit beta	↓ in SnCl_2_/NaF/AmF compared to Control (−6.02)	Calcium ion binding
Rho GDP-dissociation inhibitor 2	↓ in SnCl_2_/NaF/AmF compared to Control (−5.53)	GTPase activation

Pellicle modification with polyphenols decreased the number of exclusive proteins in the pellicles: BBE and BBE + F^−^ presented only 6 exclusive proteins (Fig. 2) each; GTE and GSE presented only 4 exclusive proteins each, and when fluoride was present in the latter groups (GTE + F^−^, and GSE + F^−^) the number of exclusive proteins was reduced to only 1 or 2 (Figs. 3 and 4).

Considering the (non-exclusive) proteins, pellicle modification with BBE or BBE + F^−^ generally caused a lower abundance of proteins (Fig. 2; Table 2). Modification with GTE caused mostly higher abundances in comparison to the NaF group, but GTE + F^−^ caused lower abundance in relation to the (control) non-modified pellicle group (Fig. 3; Table 3). Modification with GSE caused less abundance of some proteins in relation to the (control) non-modified pellicle; while GSE + F^−^ caused both lower abundance in relation to the non-modified pellicle, and higher abundance in relation to NaF and GSE (Fig. 4; Table 4).

**Table 2:** Proteins with different abundances and their biological functions in the comparison of groups: negative control, BBE, BBE + F^−^ and NaF (from Fig. 2)

Protein	Difference in abundance (log_2_ fold change)	Biological Function
Calmodulin-like protein 5	↓ in BBE + F^−^ (−6.79) compared to control, and in BBE (−7.18 and − 7.98, respectively) compared to control and NaF	Calcium ion binding
Rho-related GTP-binding protein RhoC	↓ in BBE + F^−^ (−6.42) and BBE (−6.45) compared to control	GTPase activation
Isoform 2 of Haptoglobin	↓ in BBE + F^−^ (−7.15, −6.08 and − 5.80, respectively) compared to control, NaF, and BBE	Immunity
WD repeat-containing protein 1	↓ in BBE + F^−^ (−4.96) compared to BBE	Actin-binding
Protein S100-A6	↓ in BBE + F^−^ (−7.83 and − 7.38) compared to control and NaF	Calcium ion binding
Neurofilament heavy polypeptide	↑ in BBE + F^−^ (11.80, 11.93 and 11.50, respectively) compared to control, NaF, and BBE	Cytoskeleton organization
Glutaredoxin-1	↑ in BBE + F^−^ (6.39) compared to BBE	Sodium channel regulator activity
Aspartate aminotransferase, cytoplasmic	↓ in BBE (−6.37 and − 5.25, respectively) compared to control and NaF	Aminotransferase
Protein S100-A14	↓ in BBE (−9.02 and − 7.29, respectively) compared to BBE + F^−^ and NaF	Cell survival and apoptosis
Protein S100-A16	↓ in BBE (−5.97) compared to NaF	Calcium ion binding
Ras-related C3 botulinum toxin substrate 2	↓ in BBE (−4.92 and − 5.23, respectively) compared to BBE + F^−^ and control	GTP-binding
Isoform 2 of Glucosidase 2 subunit beta	↓ in BBE (−6.88) compared to control	Calcium ion binding
Tubulin beta-2 A chain	↓ in BBE (−7.65) compared to control	GTP-binding
Kallikrein-14	↓ in BBE (−5.45) compared to control	Protease
Rho GDP-dissociation inhibitor 2	↑ in BBE (7.44) compared to NaF	GTPase activation
Hemopexin	↓ in NaF (−5.43) compared to control	Transport heme

**Table 3:** Proteins with different abundances and their biological functions in the comparison of groups: negative control, GTE, GTE + F^−^ and NaF (from Fig. 3)

Protein	Difference in abundance (log_2_ fold change)	Biological Function
Hemopexin	↓ in GTE + F^−^ (−6.45) and NaF (−5.43) compared to control	Transport heme
Kallikrein-14	↓ in GTE + F^−^ (−6.20 and − 5.61, respectively) compared to control and GTE	Protease
Alpha-amylase 2B	↓ in GTE + F^−^ (−7.90 and − 7.67, respectively) compared to control and NaF	Carbohydrate metabolism
Rho-related GTP-binding protein RhoC	↓ in GTE + F^−^ (−5.93) compared to control	GTP-binding
Isoform 2 of Glucosidase 2 subunit beta	↓ in GTE + F^−^ (−6.62) compared to control	Calcium ion binding
Tubulin beta-2 A chain	↓ in GTE + F^−^ (−7.46) compared to control	GTP-binding
Kallikrein-13	↓ in GTE + F^−^ (−5.64) compared to control	Protease
Histidine ammonia-lyase	↑ in GTE + F^−^ (5.33) compared to GTE	Histidine metabolism
Neutral alpha-glucosidase AB	↑ in GTE + F^−^ (8.23) and GTE (8.82) compared to NaF	Carbohydrate metabolic process
Immunoglobulin lambda-1 light chain	↓ in GTE (−7.20 and − 6.52, respectively) compared to control and NaF	Immunity
Isoform 2B of Desmocollin-2	↓ in GTE (−6.00 and − 5.50, respectively) compared to control and NaF	Calcium ion binding
Isoform 3 of GRB10-interacting GYF protein 2	↓ in GTE (−9.11 and − 9.77, respectively) compared to control and NaF	RNA-binding
Proteasome subunit alpha type-6	↓ in GTE (−4.75) compared to NaF	RNA-binding
Protein S100-A16	↓ in GTE (−5.58) compared to NaF	Calcium ion binding
Protein S100-A14	↓ in GTE (−8.05) compared to NaF	Cell survival and apoptosis
Neurofilament heavy polypeptide	↑ in GTE (11.29 and 11.41, respectively) compared to control and NaF	Cytoskeleton organization
Submaxillary gland androgen-regulated protein 3B	↑ in GTE (5.94) compared to NaF	Endopeptidase inhibitor activity
Histidine-rich glycoprotein	↑ in GTE (5.61) compared to NaF	Heparin-binding
Leukotriene A-4 hydrolase	↑ in GTE (7.03) compared to NaF	Metalloprotease
Rho GDP-dissociation inhibitor 2	↑ in GTE (7.61) compared to NaF	GTPase activation
Actin-related protein 3	↑ in GTE (5.19) compared to NaF	Actin-binding
Fibromodulin	↑ in GTE (5.74) compared to NaF	Collagen fibril organization
Submaxillary gland androgen-regulated protein 3 A	↑ in GTE (6.63) compared to NaF	Endopeptidase inhibitor activity
Fibulin-5	↑ in GTE (6.14) compared to NaF	Calcium ion binding

**Table 4:** Proteins with different abundances and their biological functions in the comparison of groups: negative control, GSE, GSE + F^−^ and NaF (from Fig. 4)

Protein	Difference in abundance (log_2_ fold change)	Biological Function
Actin-related protein 2/3 complex subunit 3	↓ in GSE + F^−^ (−4.83) and GSE (−4.69) compared to control	Actin-binding
Isoform 2 of Glucosidase 2 subunit beta	↓ in GSE + F^−^ (−5.58) and GSE (−7.56) compared to control	Calcium ion binding
Histone H3-7	↓ in GSE + F^−^ (−5.91) compared to control	DNA-binding
Isoform Gamma-A of Fibrinogen gamma chain	↓ in GSE + F^−^ (−6.63) compared to control	Blood coagulation
Protein disulfide-isomerase A4	↓ in GSE + F^−^ (−6.87) compared to control	RNA-binding
Isoform 2 of Histone H2B type 2-F	↓ in GSE + F^−^ (−7.71) compared to control	DNA-binding
Kallikrein-14	↓ in GSE + F^−^ (−6.26) compared to control	Protease
Apolipoprotein A-II	↓ in GSE + F^−^ (−5.45 and − 6.73, respectively) compared to control and NaF	Lipid transport
Protein S100-A12	↓ in GSE + F^−^ (−6.93 and − 8.74, respectively) compared to control and NaF	Immunity
Secreted frizzled-related protein 1	↓ in GSE + F^−^ (−6.18 and − 5.40, respectively) compared to control and NaF	Developmental protein
Isoform 2 of Cadherin-1	↓ in GSE + F^−^ (−7.07 and − 7.46, respectively) and GSE (−8.19 and − 8.58, respectively) compared to control and NaF	Calcium ion binding
High mobility group protein B2	↓ in GSE + F^−^ (−6.66 and − 5.85, respectively) and GSE (−8.40 and − 7.59, respectively) compared to control and NaF	Defense response to Gram-negative bacterium
Macrophage migration inhibitory factor	↓ in GSE + F^−^ (−5.72, −5.21 and − 5.58, respectively) compared to control, NaF and GSE	Immunity
Protein S100-A16	↓ in GSE + F^−^ (−4.38) compared to NaF	Calcium ion binding
Kallikrein-7	↓ in GSE + F^−^ (−5.61) compared to NaF	Protease
Proteasome subunit alpha type-6	↓ in GSE + F^−^ (−4.36) compared to NaF	RNA-binding
Glutaredoxin-1	↓ in GSE + F^−^ (−4.70) compared to GSE	Sodium channel regulator activity
Thrombospondin-1	↑ in GSE + F^−^ (5.72 and 7.45, respectively) compared to control and NaF	Calcium ion binding
Isoform 2 of Puromycin-sensitive aminopeptidase	↑ in GSE + F^−^ (6.44) compared to NaF	Metalloprotease
WD repeat-containing protein 1	↑ in GSE + F^−^ (6.50) compared to NaF	Actin-binding
Isoform 1 of Cell division control protein 42 homolog	↑ in GSE + F^−^ (5.77) compared to NaF	Hydrolase
Isoform 2 of 14-3-3 protein sigma	↑ in GSE + F^−^ (6.64) compared to GSE	Protein binding
Peroxiredoxin-2	↑ in GSE + F^−^ (6.74) compared to GSE	Peroxidase
Neutral alpha-glucosidase AB	↑ in GSE + F^−^ (6.97 and 10.09, respectively) and GSE (6.85 and 9.97, respectively) compared to control and NaF	Carbohydrate metabolic process
Fibulin-5	↑ in GSE + F^−^ (5.84 and 7.48, respectively) and GSE (5.96 and 7.60, respectively) compared to control and NaF	Calcium ion binding
Histidine-rich glycoprotein	↑ in GSE + F^−^ (6.65 and 7.15, respectively) compared to control and NaF, and ↑ in GSE (6.85) compared to NaF	Heparin-binding
Leukotriene A-4 hydrolase	↑ in GSE + F^−^ (7.39) and GSE (7.12) compared to NaF	Metalloprotease
Rho GDP-dissociation inhibitor 2	↑ in GSE + F^−^ (7.45) and GSE (7.10) compared to NaF	GTPase activation
Immunoglobulin heavy variable 3–15	↓ in GSE (−7.25) compared to control	Immunity
Immunoglobulin heavy constant gamma 3	↓ in GSE (−8.33) compared to control	Immunity
Immunoglobulin kappa variable 3–11	↓ in GSE (−6.19) compared to control	Immunity
Rho-related GTP-binding protein RhoC	↓ in GSE (−5.98) compared to control	GTP-binding
Kallikrein-13	↓ in GSE (−5.79) compared to control	Protease
Transthyretin	↓ in GSE (−6.38 and − 5.01, respectively) compared to control and NaF	Hormone
Isoform 3 of Acid ceramidase	↓ in GSE (−6.44) compared to NaF	Hydrolase
Fibromodulin	↑ in GSE (8.18 and 7.25, respectively) compared to control and NaF	Collagen fibril organization
Actin-related protein 3	↑ in GSE (5.91) compared to NaF	Actin-binding
Submaxillary gland androgen-regulated protein 3 A	↑ in GSE (6.07 and 7.77, respectively) compared to GSE + F^−^ and NaF	Endopeptidase inhibitor activity
Hemopexin	↓ in NaF (−5.43) compared to control	Transport heme

## Discussion

The present study mapped out, though only in vitro, the proteomic composition of modified dentine pellicles when solutions containing polyphenols and/or fluoride, or solutions containing stannous and fluoride ions, were used. Since the treatment with the solutions did modify the pellicles, leading to different compositions, the null-hypothesis was rejected.

For the two solutions containing fluoride (without polyphenols), one was based on NaF and the other on SnCl_2_/NaF/AmF. The mechanism of action of fluoride is traditionally attributed to the formation of CaF_2_-like particles. They act as a barrier, albeit weak, against acid attacks [33], though the particles do not completely cover the surface [34, 35]. The formation of these particles is strongly correlated to high fluoride concentrations and low pH [31]. Its contribution to a protective effect might be rather limited in the solutions used here, likely being highest for SnCl_2_/NaF/AmF, as it had the lowest pH and highest fluoride concentration. Stannous, on the other hand, has an additional action by incorporating into the near-surface (demineralized) layer [36]. In case of using these solutions on dentine, both F^−^ and Sn^2+^ will further act by inhibiting the matrix metalloproteinases (MMPs) [30, 37]. In this study, we now offer a fresh perspective to the mode of action of F^−^ and Sn^2+^ on the pellicle (more specifically the dentine pellicle), as we showed that F^−^ and Sn^2+^ will impact its proteomic profile.

When comparing the pellicles modified with NaF and SnCl_2_/NaF/AmF, a clear trend emerged. Although the number of exclusive proteins were similar in these groups, as well as in the control group (ranging from 12 to 15, Fig. 1), the impact on the non-exclusive proteins was that SnCl_2_/NaF/AmF caused less abundance of proteins. Interestingly, Protein S100-A6 and Glucosidase 2 subunit beta were less abundant after application of SnCl_2_/NaF/AmF, and these proteins are related to a calcium-binding mechanism. This suggests that there might be a competitive interaction between SnCl_2_/NaF/AmF and the proteins at certain calcium sites on the dentine surface, although the specifics of this interaction remain to be elucidated. Nevertheless, these results appear to indicate that fluoride and stannous ions will have an additional mechanism of action, by modifying the salivary pellicle formed on the dentine.

Similarly, polyphenols will also change proteomic profile of the pellicle, they will also inhibit MMPs [38–40] and crosslink the collagen in dentine [41], increasing the mechanical properties of the dentine and further improving their protective effect against acid attacks [38, 42, 43]. Our study showed that different polyphenols cause specific changes to the acquired pellicle, and these changes are even more evident if they are applied in combination with fluoride, especially for grape seed and green tea extracts [27, 28].

Previous studies have already shown that blending polyphenols and fluoride into mouthwash solutions will lead to an improved protective effect [29, 44]. This combination enhances the mechanism of action in many ways. On one hand, the fluoride can form, to a limited extent, CaF_2_-like particles on the dentine surface, inhibit matrix metalloproteinases (MMPs), and modify the pellicle. On the other hand, the polyphenols can crosslink the collagen of dentine, inhibit MMPs, and also modify the pellicle by crosslinking the proteins and increasing the protein-protein interaction. The latter results in a thicker and more electron-dense pellicle [4, 16, 45], which can protect against acid attacks [17, 46].

The action of polyphenols on the collagen and proteins of the pellicle is primarily due to their chemical structures. The molecules are comprised of phenol rings and galloyl esters or hydroxyl groups. These moieties enable the molecules to form complexes, particularly with salivary proteins, such as proline-rich proteins (PRP) [47] and statherin [15], which are present in the pellicle, especially at the basal layer of the enamel pellicle. For this reason, our experiment was made with an initial exposure to saliva, then the specimens were exposed to the solutions, and later more saliva was applied. This is in parallel to what happens in the clinical situation, where the teeth are almost always covered with (at least a basal layer of) an acquired pellicle [48]. Given the abundance of these proteins (PRP and statherin) in the pellicle’s basal layer, it is likely that the polyphenol molecules interact with these proteins, possibly aggregating other proteins into the pellicle. In other words, the polyphenols enhance the protein-protein interaction, thus increasing the thickness of the pellicle [17] and its ability to protect against acids [23, 24].

Looking at each polyphenol separately, we observed that they caused different modifications to the dentine acquired pellicle. This is possibly due to their individual chemical structures. For example, blueberry is rich in anthocyanins, especially malvidin-3-glucoside and malvidin-3-galactoside. A previous study [27] already showed that blueberry extract does not produce the same protective effects like green tea and grape seed extracts on dentine. The authors further suggested that these polyphenols probably do not have the same binding affinities to the proteins in the salivary pellicle or to collagen, possibly due to the configuration of the phenolic molecules [27]. Our present results seem to corroborate these speculations, since only 16 proteins in the blueberry extract groups (BBE or BBE + F^−^) showed significantly different abundances. This number is quite lower than those found for GTE/GTE + F^−^ and GSE/GSE + F^−^, where a total of 24 and 39 proteins, respectively, had different abundance. Moreover, from the 16 proteins with different abundances after pellicle modification with BBE or BBE + F^−^, 13 were significantly less abundant. We, therefore, hypothesize that the protective effect from blueberry extract, albeit lower than GTE and GSE [27], is probably due to reasons other than different abundances of proteins in the pellicles. These reasons, however, still need to be further studied.

Regarding green tea extract (GTE), previous researches have already demonstrated its beneficial effects in preventing erosion of dentine [19, 39, 49–51]. This is because GTE is rich in catechins, more specifically epigallocatechin-3-gallate (EGCG). This polyphenol can crosslink proteins in the pellicle [23, 24], leading to pellicles richer in statherin [25]. Our proteomic analysis, however, did not identify higher abundances of statherin in the modified pellicles. In fact, the majority of proteins present in the GTE groups (either with or without fluoride) had significantly lower abundances. In the GTE + F^−^ group, only two proteins were more abundant: Histidine ammonia-lyase and Neutral alpha-glucosidase AB, whose functions are rather related to histidine metabolism or carbohydrate metabolism, respectively. Therefore, even though the acquired pellicles modified with GTE have a manyfold mode of action [27], the present study could not identify the proteins involved with this mechanism.

Presently, it is known that the mechanism of GTE is rather related to the galloyl moiety present in the EGCG. This moiety is rich in hydroxyl groups that form multiple hydrogen bonds with the collagen in dentine and proteins from the pellicle, as well as with MMPs, thus inhibiting these proteases [52]. Interestingly, our results show that, in the pellicle groups modified with GTE + F^−^, there was a significantly lower abundance of Kallikrein-14 and Kallikrein-13, both with protease function, whereas Leukotriene A-4 hydrolase, with a metalloprotease function, had a significantly higher abundance. In general, Kallikreins seem to have a more general proteolytic function with trypsin-like activities at pH levels between pH 5 and pH 9. It is not clear, however, how these proteases are acting on the modified pellicles, but we can speculate that they might cleave proteins within the pellicle itself or within the dentine organic matrix. Thus, if they are less abundant in the modified pellicle groups, the pellicle and dentine collagen will be less degraded, and this possibly elucidates one of the protective effects of the polyphenols/fluoride [27, 29]. On the other hand, the higher abundance of Leukotriene A-4 hydrolase may not be affecting this mode of action, because this metalloprotease has a more specific function of catalysing Leukotriene A4 to Leukotriene B4. So even though this metalloprotease is more abundant in the modified pellicle, it is not having any degradative effect on the proteins of the pellicle or on the collagen layer of dentine.

Grape seed extract (GSE) also exhibits comparable protective effects, both on enamel and on dentine [27–29, 38, 43, 44]. Our GSE solutions are rich in oligomeric proanthocyanidin (OPC), a type of polyphenol made up of catechin and epicatechin units with partial galloylation [53]. This composition allows OPC to engage in hydrophobic interactions, hydrogen bonding with proteins, and oxidation that lead to covalent bonding [47, 54, 55] with other molecules. In case of the acquired pellicle, OPC readily binds to PRP and statherin [15], thus improving the pellicle’s protective function [23, 24], both on enamel [19] and on dentine [27]. Furthermore, similarly to GTE, the solutions containing GSE (with or without fluoride) caused a significantly lower abundances in proteases, namely Kallikrein-14, Kallikrein-7, and Kallikrein-13. Again, the lower abundance of these proteases in the pellicles modified with GSE may also partially elucidate the mechanism of action of OPC on the acquired pellicle, that these proteases with trypsin-like activity will be present in fewer numbers and, thus, will cause less cleavage activity to the proteins of the pellicle and collagen. This, in turn, will lead to better protection against dentine demineralization. Similarly, to the GTE groups, GSE and GSE + F^−^ also caused higher abundance of some metalloproteases (Leukotriene A-4 hydrolase and Isoform 2 of Puromycin-sensitive aminopeptidase), but again, these have more specific proteolytic functions, and they are probably not playing any negative role in the mechanism of action of GSE.

When the plant extracts were combined with NaF, a more clear-cut difference was observed for GTE and GSE. When the pellicles were modified solely with NaF, around 10–14 proteins were observed exclusively in these pellicles. Likewise, a similar number of exclusive proteins were observed in the non-modified pellicles. Interestingly, when the pellicles were modified with polyphenols, the number of exclusive proteins found in the pellicles dropped to around 4 to 6. This number dropped even further when the pellicles were modified with GTE + F^−^ and GSE + F^−^, where the only proteins exclusive to these modified pellicles were Protein POF1B and/or Isoform 2 of Puromycin-sensitive aminopeptidase. Interestingly, despite this lower numbers of exclusive proteins in the modified pellicles, the combination of fluoride and polyphenols (like in GTE + F^−^ and GSE + F^−^) still show a synergistic protective effect [28, 29, 44], and this lower number of proteins may not be influencing the beneficial effects of the pellicle modification. Interestingly, a similar synergistic effect has also been observed when proteins were combined with fluoride for pellicle modification [56]. The details of how these synergisms between organic (polyphenols or proteins) and inorganic (fluoride) compounds occur still need to be further studied.

Although the present study maps out the proteomic differences between the different modified pellicles, the exact effect of the different protein-protein interactions in the modified pellicles are still far from being well-understood. More research is still necessary to determine the roles of the different proteins in the pellicle, both on enamel as well as on dentine, and how they affect demineralization. A limitation of the present study is also that we had no verification of the complete removal of the pellicles. Although we did follow established protocols, there is evidence that parts of the basal layer remain at the surface even after scraping off the (enamel) pellicle [57]. On the other hand, there is conflicting evidence whether the dentine pellicle has a basal layer [11], and the pellicles were all harvested in a standardized way, allowing for at least the comparison of the pellicles within this study. The differences between in vitro and clinical pellicles may also lead to differences in the mode of action of these solutions in vivo, so a direct extrapolation to in vivo conditions should be made with great care. Still, at present, we can only conclude that a solution containing 500 ppm F^−^, either in the form of NaF or as SnCl_2_/NaF/AmF, has a significant modifying effect on the proteome of the dentine pellicle. From which we suggest that F^−^ and Sn^2+^ not only act on the tooth mineral, but they will also have a supplementary mode of action – acting on the acquired pellicle. We further conclude that polyphenols alone, and especially in combination with fluoride, can modify the proteomic composition of the dentine pellicles, lowering the abundance of some proteins and proteases, which further elucidates, in parts, how these compounds protect dentine against demineralization.

## Data Availability

The data that support the findings of this study are available from the corresponding author upon reasonable request.
